# In-line sample concentration in capillary electrophoresis by cyclodextrin to admicelle microextraction

**DOI:** 10.1007/s00216-022-04230-0

**Published:** 2022-08-18

**Authors:** Andaravaas Patabadige Jude P. Vaas, Raymond B. Yu, Joselito P. Quirino

**Affiliations:** 1 Australian Centre for Research On Separation Science (ACROSS), School of Natural Sciences-Chemistry, University of Tasmania, Private Bag 75, Hobart, TAS 7001 Australia; 2 Present Address: Department of Pharmaceutical Chemistry, College of Pharmacy, University of the Philippines Manila, Manila, Philippines

**Keywords:** Capillary zone electrophoresis, Cyclodextrin, Electroosmotic flow, Microextraction, Pseudophase, Sample concentration

## Abstract

Cyclodextrins (CDs) as a pseudophase in pseudophase-to-pseudophase microextraction (P^2^ME) in capillary zone electrophoresis (CZE) are proposed. In this P^2^ME mode called CD to admicelle ME, a long plug of dilute analyte solution prepared in cetyltrimethylammonium bromide (CTAB) at the critical micellar concentration was injected into the capillary. This formed CTAB admicelles at the interface between the solution and the negatively charged capillary surface, where the analytes were trapped. The injection of CD solution released the admicelles and the analytes from the capillary surface due to the formation of stable CD/CTAB inclusion complexes. The analytes are concentrated at the CD front during injection and voltage separation. Various neutral CDs were found to be effective for CD to admicelle ME. To implement this in-line sample concentration technique in CZE, CD concentration, sample injection time, and sample:CD solution injection ratio were optimized. The optimized conditions for five model anionic analytes, namely, 4-bromophenol, sulindac, sulfamethizole, 4-vinylbenzoic acid, and succinylsulfathiazole, were 20 mM α-CD in 20 mM sodium tetraborate (pH 9.2) solution, sample injection time of 370 s, and CD:sample injection ratio of 1:2. The sensitivity enhancement factors (SEFs) were between 112 and 168. The SEFs of sulindac and sulfamethizole in particular were similar to previously published off-line microextraction techniques, which are typically time-consuming. The calculated values of LOQ, intra-/inter-day (*n* = 6/*n* = 10, 3 days) repeatability, and linearity (*R*^2^) of CD to admicelle ME were 0.0125–0.05 µg/mL, 1.5–4.6%, 1.8–4.8%, and ≥0.999, respectively. Finally, the potential of CD to admicelle ME to the analysis of artificial urine samples was demonstrated.

**Supplementary Information:**

The online version contains supplementary material available at 10.1007/s00216-022-04230-0.

## Introduction

Capillary electrophoresis (CE) is a family of microscale analytical separation techniques which utilizes the electric field to separate analytes inside a narrow-inner diameter (id) fused silica capillary [1–6]. The separation of neutral or charged small or large molecules is done by the appropriate choice of the CE separation media. In the CE mode of capillary zone electrophoresis (CZE), the separation media or background solution (BGS) is a buffer. Tuning the pH of the BGS used in CZE typically allows the efficient separation of charged species due to differences in their electrophoretic mobilities. CZE is popularly used in the analysis of charged small molecules in commercial drug/herbal products and biological samples [7–16] and food/beverages and environment [17–25].

A well-known limitation in CE is poor detection sensitivity with UV detection. From the Beer’s law, the analyte absorbance is directly related to the concentration and pathlength, which is equal to the id of a capillary. The concentration sensitivity of CE-UV with a typical 50 µm id capillary can be two orders of magnitude poorer when compared to liquid chromatography (with off-line UV detection). Dedicated off-line sample concentration steps were often developed to achieve fit-for-purpose CE-UV assays for various applications [14, 26, 27]. The development of in-line sample concentration or stacking techniques in CE is also an active area of research, with 30–70 papers published each year during the last decade [28–32]. The popularity of stacking as an analyte concentration approach is attributed to its general ease of implementation and effectiveness.

In typical injection CE, samples are normally prepared in the separation media to achieve sharp peaks. However, stacking CE produces sharp peaks even with longer sample injections. This is achieved by the appropriate manipulation of sample solution chemistry and injection parameters. The analytes in the long plug are focused into a narrow zone in the presence of an electric field. Established in-line sample concentration techniques that are purely based on electrophoretic effects include stacking by field amplification/enhancement [33, 34], transient isotachophoresis [35–38], and dynamic pH junction [39, 40]. Stacking techniques of sweeping, analyte focusing by micelle collapse, and micelle to solvent or cyclodextrin stacking [41–45] also utilize the interaction of the analytes with a pseudophase (e.g., micelles). In the new in-line sample concentration technique in CZE called electroosmotic flow (EOF) assisted pseudophase-to-pseudophase microextraction (P^2^ME), a long plug of a dilute solution of analytes (e.g., 12.4 cm) prepared in a solution of cetyltrimethylammonium bromide (CTAB) (where [CTAB] was between critical surface aggregation concentration (csac), and critical micellar concentration (cmc)) was injected into the capillary [46]. The analytes trapped in the CTAB stationary pseudophase were released and concentrated by the introduction of another pseudophase (i.e., sodium dodecyl sulfate (SDS) micelles). The SDS micelles were partially introduced into the capillary by pressure. Upon application of voltage, the EOF generated pushed the negatively charged micelles towards the cathode. Sensitivity enhancement factors (SEFs) in the order of 10 were achieved using this technique.

Long-chain ionic surfactants (e.g., SDS and CTAB) form interfacial and solution micelles at concentrations > cmc [47]. At concentrations > csac, these surfactants form admicelles (surfactant bilayer) at the interface between the liquid and capillary wall surface (with opposite charge) [48], which was demonstrated previously in CE [48, 49] and optical refractometry [50] studies. These surfactant aggregates at the interface have been shown to act as chromatographic pseudophases for open-tubular separations [48, 51–54]. Moreover, surfactant aggregates (i.e., admicelles) have recently been proposed for in-line sample concentration via pseudophase microextraction in CE [55].

Here, we used a neutral pseudophase, i.e., cyclodextrins (CD), instead of SDS micelles in P^2^ME (CD to admicelles ME) of anionic analytes in CZE. The analytes are released from the CTAB admicelles and concentrated by the formation of stable CD/CTAB inclusion complexes, which increase the csac of CTAB, thus collapsing the admicelles [56, 57]. P^2^ME parameters such as nature and concentration of CD, sample, and CD plug injection time were systematically investigated. Analytical figures of merit (linearity, repeatability, limits of quantitation (LOQ)) were determined. The potential application of P^2^ME to fortified artificial urine was also explored.

## Materials and methods

### Reagents, solutions, and sample

HPLC-grade acetonitrile (ACN) and methanol (MeOH), sodium hydroxide (NaOH), sodium tetraborate, CDs (α-CD, β-CD, γ-CD, carboxymethyl-β-CD (CM-β-CD), hydroxyethyl-β-CD (HE-β-CD), hydroxypropyl-α-CD (HP-α-CD), hydroxypropyl-β-CD (HP-β-CD), hydroxypropyl-γ-CD (HP-γ-CD), γ-CD phosphate sodium salt (P-γ-CD), sulfated α-CD (S-α-CD), and sulfated γ-CD (S-γ-CD)), CTAB, and artificial urine were either from Sigma-Aldrich (St. Louis, MA) or Fluka (Buchs, Switzerland). Solutions were prepared in ultrapure water which was obtained from a Milli-Q system (Millipore, Bedford, MA, USA). All solutions and samples were sonicated for ~ 1 min and then passed through a syringe filter with a pore diameter of 0.45 µm (Agilent Technologies, Waldbronn, Germany) prior to use. The BGS was 20 mM sodium tetraborate (pH 9.2) with 10% (v/v) ACN. The CD solution was 0.05–20 mM of a CD in 20 mM sodium tetraborate (pH 9.2). Model anionic analytes (2 mg/mL) (4-bromophenol, 4-vinylbenzoic acid, dichlorprop, ibuprofen, indoprofen, succinylsulfathiazole, sulfamerazine, sulfamethizole, sulfaquinoxaline, sulindac) were prepared in MeOH and were stored in 4 °C until use. In typical injection, the analytes were prepared in BGS. In P^2^ME, the analytes were prepared in 0.2 mM CTAB in 20 mM sodium tetraborate (pH 9.2) (P^2^ME sample diluent).

Artificial urine was processed according to the method of Wildman et al. [58]. Briefly, artificial urine (8 µL) was spiked with the standard analyte mixture and was diluted 1:50 (v/v) in 10% ACN in 20 mM sodium tetraborate. CTAB was then added to obtain a sample matrix with a final concentration of 0.2 mM CTAB in 20 mM sodium tetraborate and an analyte concentration of 0.4 µg/mL.

### CZE instrumentation and general procedure

CZE was performed using an Agilent HPCE (Waldbronn, Germany) equipped with a UV detector and fitted with a fused silica capillary (Polymicro Technologies, Phoenix, AZ) with od and id of 360 µm and 50 µm, respectively. The total length of the capillary was 37.5 cm or 50 cm (29 cm or 41.5 cm from the inlet to the detector at 200 nm, respectively). The capillary temperature was maintained at 20 °C.

New capillaries were conditioned at ~ 1000 mbar with 0.1 M NaOH for 10 min followed by purified water for 5 min. Prior to each run, the capillaries were conditioned sequentially at ~ 1000 mbar with MeOH (2 min), ultrapure water (1 min), 0.1 M NaOH (7 min), ultrapure water (1 min), and BGS (6 min). Briefly, we studied the effect of injection regimens of 25 mbar at 4 s and 50 mbar at 5, 10, and 15 s using the BGS as sample diluent. The peak heights were similar under all these conditions. However, peak broadening was observed at 5 s using 50 mbar injection pressure. Therefore, for typical injections, the sample was injected at 25 mbar for 4 s. For CD to admicelles ME, the sample was injected at 50 mbar followed by the CD plug, at various times. A separation voltage of 20 kV was applied at positive polarity (cathode at the detector end).

### Calculations

SEF was calculated using the following formula: SEF = (peak height obtained from CD to admicelles ME)/(peak height obtained from typical injection) × dilution factor.

% Recovery of analytes from the artificial urine sample was calculated using the following formula: % Recovery = (peak height from fortified sample)/(peak height from a standard analyte mixture at the same concentration) × 100.

## Results and discussion

### General procedure and mechanism of CD to admicelle ME

**Fig. 1:**
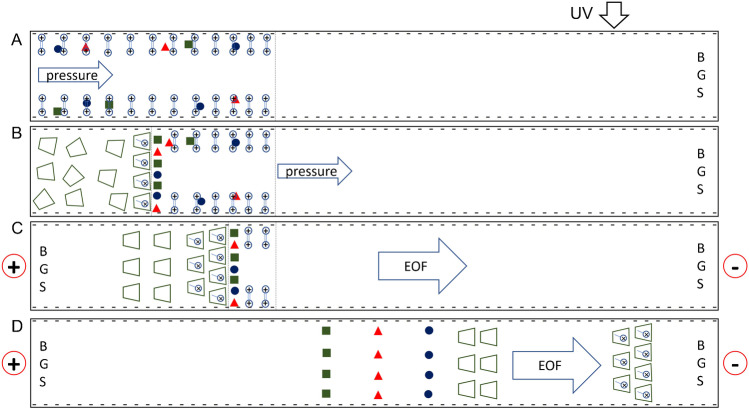
Schematic of P^2^ME (cyclodextrin to admicelle ME). A long plug of diluted analyte solution prepared in a CTAB solution (csac < [CTAB] < cmc) is injected into the capillary, with the analytes trapped in the CTAB chromatographic pseudophase (**A**). A short CD plug is then hydrodynamically injected into the capillary. A CD/CTAB complex is formed at the CD front, releasing and concentrating trapped analytes at the CD/S boundary (**B**). Voltage was applied at both ends of the capillary, with the cathode at the detector side (**C**). The EOF generated pushed the CD to the detector side, sweeping the remaining CTAB pseudophase and releasing the remaining analytes. Finally, the stacked analytes are separated by CZE (**D**)

The general procedure and mechanism of CD to admicelles ME are shown in Fig. 1. After conditioning the capillary with 0.1 M NaOH (flushed with purified water before and after) to ionize the silanol group, a long plug of a dilute analyte solution (S) prepared in a CTAB solution (csac < [CTAB] < cmc) was hydrodynamically injected into the capillary (see Fig. 1A). CTAB admicelles formed in the negatively charged capillary surface trapped the injected analytes. Then, a CD plug was hydrodynamically injected into the capillary (see Fig. 1B). The formation of CD/CTAB complexes in solution facilitated the release of the trapped analytes and the accumulation of analytes at the CD plug front. Upon application of voltage with the BGS on both ends of the capillary (see Fig. 1C), the cathodic EOF dragged the neutral CDs to the detector. The CDs swept through the remaining CTAB admicelles, causing the complete release and concentration of analytes. Finally, the released and concentrated analytes were separated by CZE (see Fig. 1D).

### Proof of concept of CD to admicelle ME

**Fig. 2:**
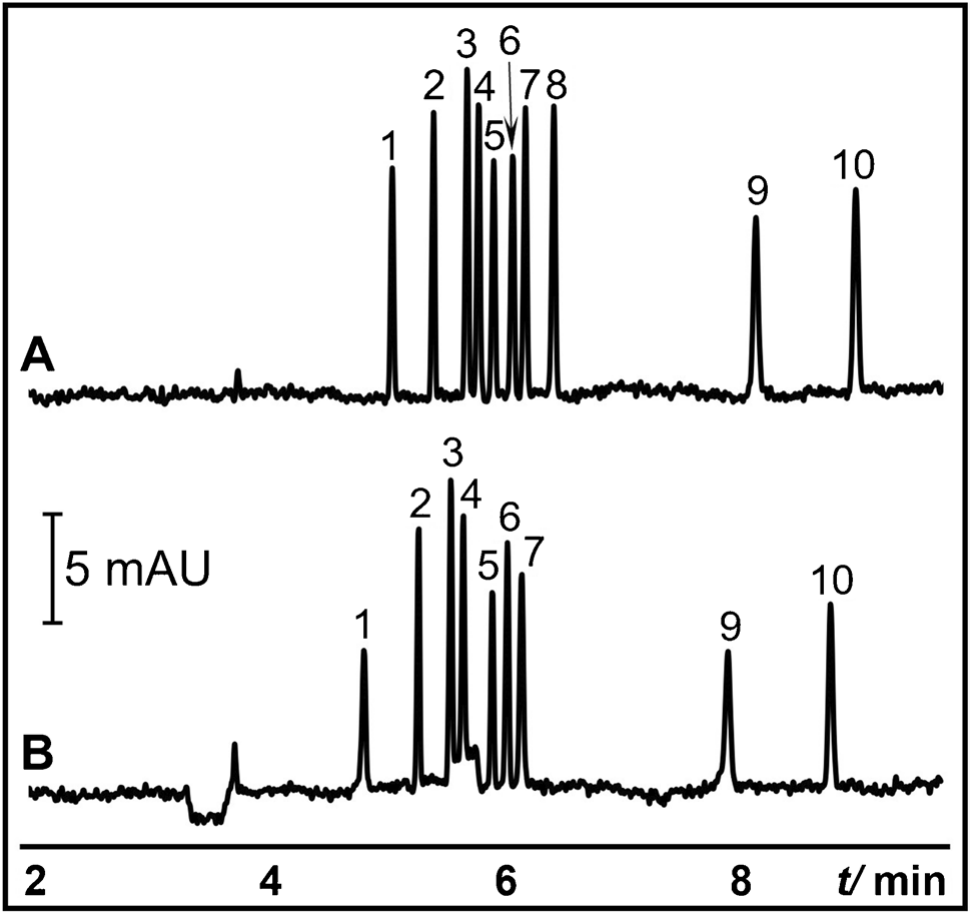
Proof of concept for P^2^ME (CD to admicelle ME) of anionic analytes. **A** is the typical injection CZE and is shown here for comparison. **B** is P^2^ME (CD to admicelle ME) injection. BGS was 20 mM sodium tetraborate (pH 9.2) with 10% ACN. Analytes were 4-bromophenol (1), sulindac (2), sulfaquinoxaline (3), indoprofen (4), sulfamerazine (5), ibuprofen (6), sulfamethizole (7), dichlorprop (8), 4-vinylbenzoic acid (9), and succinylsulfathiazole (10). For typical injection, analytes (40 µg/mL) were prepared in BGS. For P^2^ME, analytes (4 µg/mL) were prepared in 0.2 mM CTAB in 20 mM sodium tetraborate (pH 9.2). Typical injection was performed at 25 mbar for 4 s. P^2^ME injection program was as follows: 0.2 mM CTAB in borate buffer (50 mbar for 20 s), sample in 0.2 mM CTAB in borate buffer (50 mbar for 20 s), and CD plug (50 mbar for 10 s). The CD plug was 20 mM α-CD in borate buffer. Capillary dimensions were 50 cm (41.5 cm from inlet to UV detector) × 50 µm i.d. Other conditions are mentioned in the Materials and methods

The electropherograms obtained from typical injection and the proposed P^2^ME of ten anionic analytes are shown in Fig. 2A and B, respectively. Analytes in P^2^ME with concentrations 10 × lower than in typical injection were prepared in P^2^ME sample diluent. The analytes were injected into the capillary at 50 mbar for 20 s. For this demonstration, a short plug of P^2^ME sample diluent (50 mbar for 20 s) was needed to improve the enrichment. We note that for longer S injections, injection of P^2^ME sample diluent is not required. Then, a plug of 20 mM α-CD in 20 mM sodium tetraborate (pH 9.2) was injected at 50 mbar for 10 s. In this preliminary experiment, SEFs using peak heights of 6 for 4-bromophenol; 8 for sulfamerazine, sulfamethizole, and 4-vinylbenzoic acid; 9 for sulindac, sulfaquinoxaline, indoprofen, and succinylsulfathiazole; and 10 for ibuprofen were achieved. Dichlorprop peak did not appear in P^2^ME (see Fig. 2B), probably because a long injection of dichlorprop is required for its preconcentration in pseudophase microextraction [55]. We also note the faster migration time of the analytes in Fig. 2B, which was due to the shorter effective length for analysis after CD to admicelles ME. In addition, with longer sample injections (e.g., > 200 s at CD to S injection plug ratio of 1:2), stacking was incomplete, and co-elution of analyte peaks was observed due to a shorter effective separation length.

**Fig. 3:**
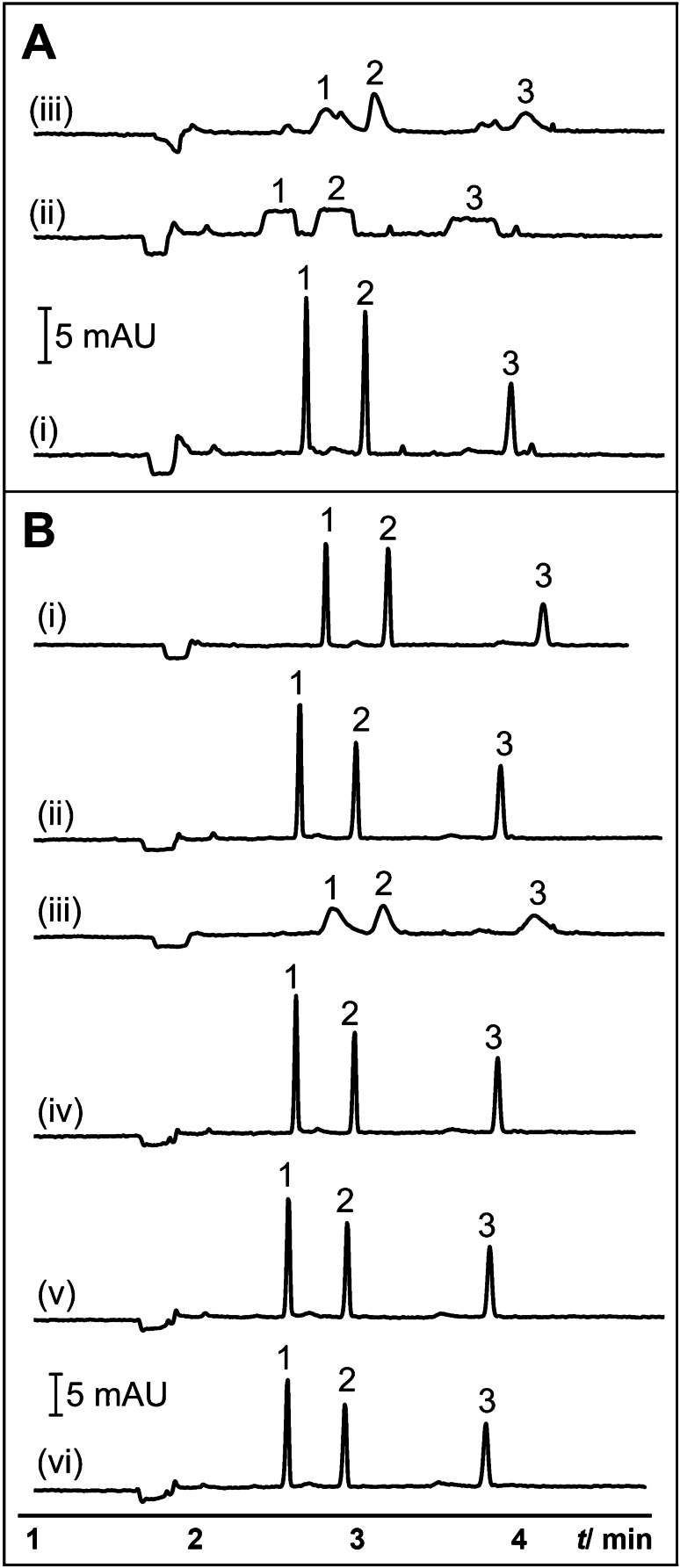
**A** Elucidation of the mechanism of P^2^ME (CD to admicelle ME). (i) was P^2^ME injection. In (ii), model anionic analytes prepared in 20 mM sodium tetraborate (pH 9.2) was injected at 50 mbar for 20 s followed by 20 mM α-CD in borate buffer at 50 mbar for 3 s. In (iii), 20 mM α-CD in borate buffer was injected at 50 mbar for 3 s, followed by anionic analytes prepared in P^2^ME sample solution at 50 mbar for 20 s. Capillary dimensions were 37.5 cm (29 cm from inlet to UV detector) × 50 µm i.d. **B** CD to admicelle ME using various native and neutral derivatized CDs. The CDs were 2 mM of α-CD (i), β-CD (ii), γ-CD (iii), hydroxypropyl-α-CD (iv), hydroxyethyl-β-CD (v), and 2-hydroxypropyl-β-CD (vi) in 20 mM sodium tetraborate (pH 9.2). P^2^ME injection program was as follows: sample injection at 50 mbar for 20 s, followed by injection of CD plug at 50 mbar for 10 s. Capillary dimensions were 50 cm (41.5 cm from inlet to UV detector) × 50 µm i.d. Model anionic analytes in **A** and **B** were 4 µg/mL each of sulindac (1), sulfamethizole (2), and 4-vinylbenzoic acid (3) in P^2^ME sample solution. BGS is as Fig. 2. Other conditions are mentioned in the Materials and methods

To prove the role of CTAB in P^2^ME, S was prepared in 20 mM sodium tetraborate (pH 9.2). To simplify the analysis, three analytes, namely, sulindac, sulfamethizole, and 4-vinylbenzoic acid, were used. S was injected at 50 mbar for 20 s, followed by the α-CD plug at 50 mbar for 3 s. Broad, plateau peaks were obtained in the absence of CTAB in S as shown in the electropherogram at Fig. 3A(ii), indicating the necessity of CTAB for sample concentration. The electropherogram showing S prepared in P^2^ME sample diluent is shown in Fig. 3A(i) for comparison. To confirm the role of EOF in P^2^ME, the α-CD plug was injected first into the capillary before the S prepared in P^2^ME sample diluent. The injection lengths of both S and the α-CD plug were as in Fig. 3B. The electropherogram in Fig. 3A(iii) did not reveal any sharp peaks, indicating that the CTAB admicelles remained attached to the negatively charged capillary wall.

### Different CDs in CD to admicelle ME

We investigated different types of CDs, including native (α-, β-, γ-CD), neutral derivatized (HE-β-CD, HP-α-CD, HP-β-CD), and anionic (CM-β-CD, P-γ-CD, S-α-CD, S-γ-CD)) CDs for implementation in CD to admicelles ME. The results are shown in Fig. 3B. The CD solution was 2 mM of a CD in 20 mM sodium tetraborate (pH 9.2). The S and CD plug were injected at 50 mbar for 20 and 10 s, respectively. No variations in peak heights were observed across the native and neutral derivatized CDs except for γ-CD (see Fig. 3B(iii)). Poor stacking was observed when γ-CD was used for CD to admicelle ME. This was attributed to the lower binding strength of γ-CD to CTAB [59], which caused inefficient analyte release and concentration in the method. On the other hand, the anionic CDs showed lower peak heights than the neutral CDs (see SI Fig. S1). Since the interaction of anionic CDs has been shown to be stronger than neutral CDs for long-chain cationic surfactants [56, 57], the strength of the interaction of an anionic CD with CTAB should be sufficient to release the admicelles from the capillary wall. On the other hand, we speculate that an anionic CD layer on top of the CTAB admicelles was formed by electrostatic interaction. This caused the inefficient release and enrichment of the anionic analytes.

### Effect of CD concentration

To instantly show the effect of neutral [CD] on sample concentration, we used 20 mM or 10 × more concentrated CD compared to Fig. 3B. Due to solubility issues, β-CD was not included. For HE-β-CD, HP-α-CD, and HP-β-CD, the concentration effect for the analytes used in Fig. 3B using a 20 mM CD was similar to a corresponding 2 mM CD. Meanwhile, the use of 20 mM α-CD or γ-CD caused sample concentration. We then selected α-CD to systematically study the effect of [CD]. The [α-CD] was varied at 0.05, 0.1, 2 and 20 mM (see SI Fig. S2). As expected, [CD] was important for efficient CD to admicelle ME. Sample concentration was poor at the lowest concentration studied, while increasing [CD] up to 20 mM improved sample concentration.

### Optimization of injection conditions

The injection ratio of CD solution and S and the S injection time were optimized using 20 mM α-CD as the CD solution. The model analytes were 4 µg/mL each of 4-bromophenol, sulindac, sulfamethizole, 4-vinylbenzoic acid, and succinylsulfathiazole. The injection ratios of 1:20, 1:4, and 1:2 (CD plug length:S plug length) were studied at a fixed S injection time of 20 s (see SI Fig. S3). Stacking was already evident at an injection ratio of 1:20. Increasing the injection ratio from 1:20 to 1:4 improved analyte peak heights. This suggested the importance of the injection time of the CD plug in CD to admicelles ME. However, increasing the injection ratio from 1:4 to 1:2 did not improve further analyte peak heights. We note that the length of S was short at ~ 1.56 cm; thus, increasing the injection ratio from 1:4 to 1:2 did not do much to improve analyte peak heights.

**Fig. 4:**
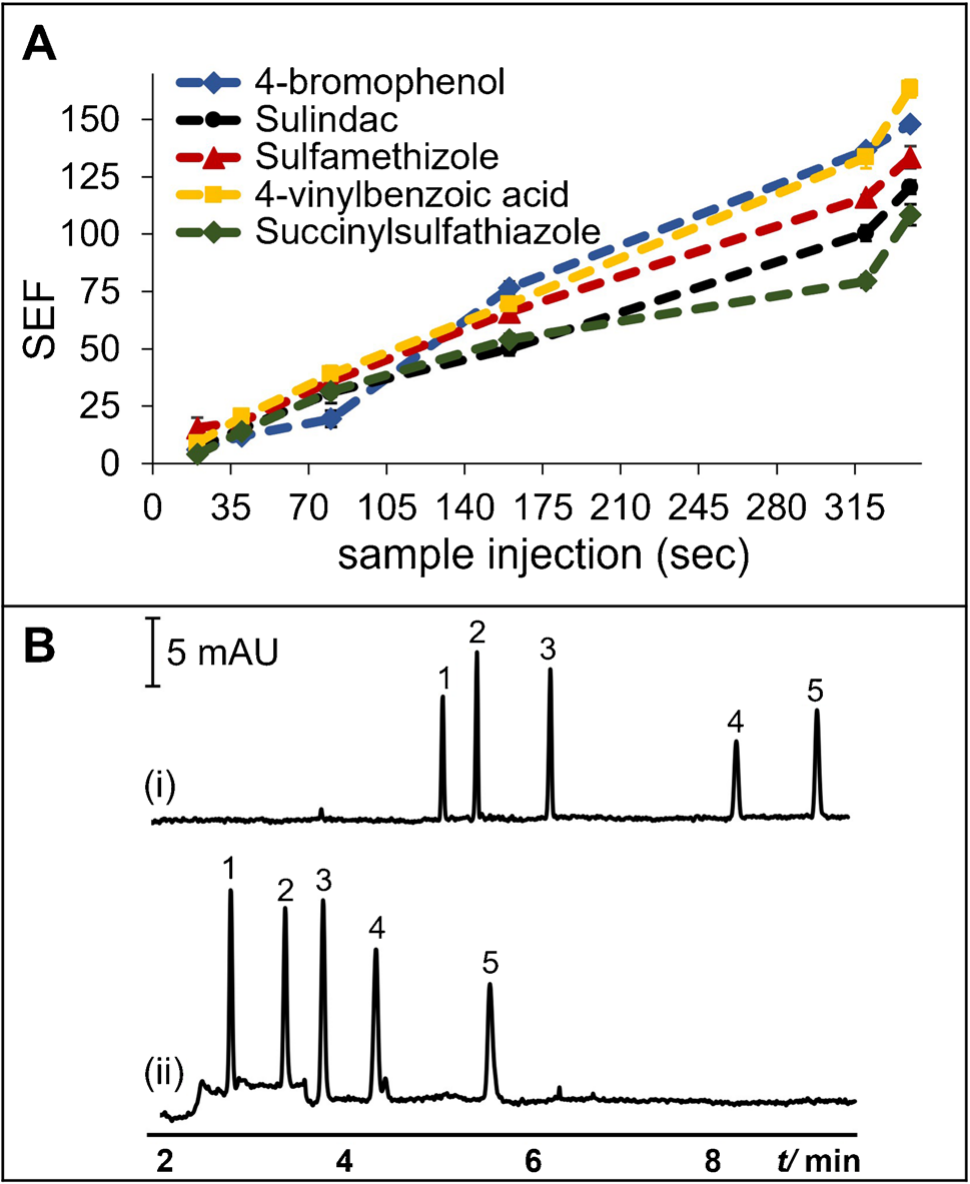
**A** Effect of sample injection time on SEF. Model anionic analytes (0.4 µg/mL) were prepared in 0.2 mM CTAB in borate buffer. BGS and CD plug were as Fig. 2. Sample and CD plug injection were performed at 50 mbar. The time of the CD plug injection was one-half of sample injection time. Other conditions are mentioned in the Materials and methods. **B** Representative electropherograms of typical injection (i) and optimized P^2^ME (CD to admicelle ME) (ii) of model anionic analytes. Typical injection was as Fig. 2. P^2^ME (CD to admicelle ME) injection program was as follows: sample injection at 50 mbar for 340 s (plug length ~ 39.7 cm), followed by injection of CD plug (20 mM α-CD in borate buffer) at 50 mbar for 170 s. Analytes were 40 (typical injection) or 0.4 (CD to admicelles ME) µg/mL each of 4-bromophenol (1), sulindac (2), sulfamethizole (3), 4-vinylbenzoic acid (4), and succinylsulfathiazole (5). Capillary dimensions were 50 cm (41.5 cm from inlet to UV detector) × 50 µm i.d. Other conditions are mentioned in the Materials and methods

The S injection times of 20–200 s were studied at injection ratios of 1:4 and 1:2. The plot of SEF vs. injection time are shown in SI Figs. S4 and S5, respectively. In general, the SEFs increased with the increase in S injection time regardless of the injection ratio. However, the concentration of 4-bromophenol was 5 × better with the 1:2 ratio at 200 s. At 200 s injections, the SEFs were 15–60 and 40–70 for the 1:4 and 1:2 ratios, respectively. At a fixed S plug length, the effective separation length at 1:2 is shorter than 1:4 because of the longer CD plug. Therefore, the stacked analytes in the 1:2 ratio were nearer the detector at the end of the stacking process. The contribution of broadening by diffusion in the 1:2 ratio was lesser than 1:4, causing slightly better SEF values in the 1:2 injection. Using the 1:2 injection ratio, we then extended the S injection from 200 to 340 s (39.7 cm) (170 s CD plug). The results are shown in Fig. 4A. After comparison with typical injection (Fig. 4B), the SEFs of 150, 123, 138, 168, and 112 for 340 s were obtained for 4-bromophenol, sulindac, sulfamethizole, 4-vinylbenzoic acid, and succinylsulfathiazole, respectively. Please note that the analytes in P^2^ME was 100 × more dilute than in typical injection CZE. The SEFs obtained for sulindac and sulfamethizole were comparable to those obtained from microextraction techniques reported in literature (see Table 1). Unfortunately, we were not able to obtain similar data for 4-bromophenol, 4-vinylbenzoic acid, and sulfamethizole in literature.

**Table 1:** Comparison of CD to admicelle ME with other microextraction techniques in literature for the tested analytes

Analyte	Method	Matrix	Sample pre-treatment time	Detection	Linear range	Limit of quantitation	Enhancement factor	Ref
Sulindac	Mixed matrix membrane tip extraction	Wastewater	~ 40 min	UHPLC-MS/MS	0.30–500 ng/L	0.30 ng/L	208	[60]
	Ultrasound-assisted dispersion liquid–liquid microextraction	Various water samples	21 min + evaporation	UHPLC-MS/MS	0.2–100 ng/mL	0.148 ng/mL	49	[61]
	CD to admicelle ME	Artificial urine	8.5 min	CZE-UV	50–800 ng/mL	50 ng/mL	123	This method
Sulfamethizole	Polymer monolith microextraction	Milk/egg	20 min	HILIC-MS	20–2000 ng/g	16.7–32.8 ng/g	8.9	[62]
	CD to admicelle ME	Artificial urine	8.5 min	CZE-UV	12.5–800 ng/mL	12.5 ng/mL	138	This method

### Analytical figures of merit

Using the conditions in Fig. 4B(ii), the LOQ, linearity (including linear range, equation of line, coefficient of variation), and intra- and inter-day repeatability in terms of corrected peak areas and peak migration times are summarized in Table 2. The LOQs (S/N = 10) of the method for the five analytes were between 0.0125 and 0.05 µg/mL. Linear range is more than one order of magnitude for all analytes, except for succinylsulfathiazole which is more than two orders of magnitude. Good linearity for the method was observed for all analytes, with *R*^2^ values > 0.990. Repeatability was determined at 8–32 × the LOQ of the analytes. The intra-day (*n* = 6) repeatability of analyte migration times and analyte peak heights were 4.6% and 2.7%, respectively. The inter-day (*n* = 10, 3 days) repeatability of analyte migration times and analyte peak heights were 4.7% and 3.3%, respectively.

**Table 2:** Analytical figures of merit of CD to admicelle ME for the analysis of five anionic analytes

Parameter	4-Bromophenol	Sulindac	Sulfamethizole	4-Vinylbenzoic acid	Succinylsulfathiazole
Linear range (µg/mL)	0.025–0.8	0.05–4.0	0.0125–0.8	0.025–0.8	0.05–4.0
Equation of the line					
*slope* ± *% RSD*					
Based on peak height	26.531 ± 1.349	28.275 ± 1.220	27.365 ± 0.504	18.311 ± 3.881	13.024 ± 0.190
Based on corrected peak area	20.871 ± 0.664	22.665 ± 0.440	21.403 ± 0.268	17.297 ± 1.087	15.384 ± 0.979
*y-intercept* ± *% RSD*					
Based on peak height	0.856 ± 6.017	0.690 ± 1.926	0.161 ± 8.847	0.034 ± 2.033	0.193 ± 2.458
Based on corrected peak area	0.395 ± 0.197	− 1.047 ± 7.627	0.369 ± 2.395	− 0.572 ± 10.097	− 1.796 ± 8.236
Coefficient of variation (*R*^2^)					
Based on peak height	0.9992	0.9998	0.9998	0.9965	0.9997
Based on corrected peak area	0.9998	0.999 8	0.9997	0.9981	0.9960
LOQ (µg/mL)	0.025	0.05	0.0125	0.025	0.05
*Intra-day repeatability (n* = 10)*					
% RSD, corrected peak area	3.4	3.0	4.6	4.0	3.2
% RSD, peak height	4.1	4.2	4.1	4.3	4.6
% RSD, migration time	1.5	1.6	1.5	2.5	2.7
*inter-day repeatability (n* = 10, 3 days)*					
% RSD, corrected peak area	4.6	4.7	4.8	4.5	4.6
% RSD, peak height	3.6	4.1	4.7	4.5	4.6
% RSD, migration time	1.8	2.1	3.2	3.3	3.2

^*^The analyte concentration used to estimate RSDs was 0.4 µg/mL

### Potential application of CD to admicelle ME to analysis of fortified artificial urine

**Fig. 5:**
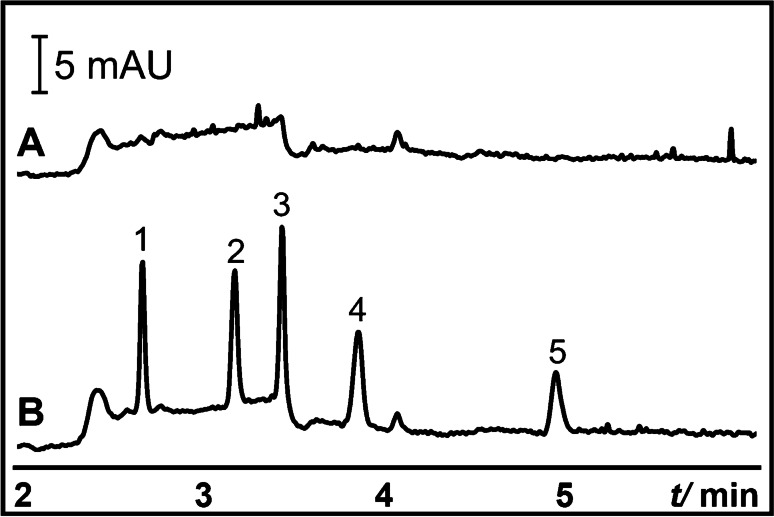
Potential application of P^2^ME (CD to admicelle ME) to the analysis of artificial urine samples fortified with 0.4 µg/mL of each analyte. Analyte identification and concentration and optimized P^2^ME (CD to admicelle ME) conditions were as in Fig. 4B(ii). Other conditions are mentioned in the Materials and methods

CD to admicelle ME-CZE was applied to the analysis of diluted and fortified artificial urine, which mimics real urine samples. The electropherograms of the unfortified and fortified artificial urine are shown in Fig. 5. No significant interferences from the sample matrix were found. Analyte recoveries for 4-bromophenol, sulindac, sulfamethizole, 4-vinylbenzoic acid, and succinylsulfathiazole were 94.7, 96.0, 114.9, 89.1, and 68.6% with %RSD (*n* = 3) values of 1.9, 1,0, 10.7, 7.2, and 0.2%, respectively. The results demonstrate the potential of the approach for the determination of small organic molecules in real samples such as urine.

## Conclusion

The use of CDs as pseudophase in P^2^ME was successfully demonstrated with significant SEFs of 112–168. The proposed technique called CD to admicelle ME is a novel in-line sample concentration approach that combines microextraction and stacking in one capillary. CD to admicelle ME is a green, easy to perform, and tuneable stacking/microextraction method and requires only the use of readily available reagents which form a chromatographic stationary pseudophase in situ. In this technique, CDs, which form stable CD/CTAB inclusion complexes, facilitated the release and concentration of the trapped analytes retained in the admicelles. Neutral CDs, i.e., α-CD, HE-β-CD, HP-α-CD, and HP-β-CD, were equally effective for this purpose. [CD], S injection times and CD:S ratio were critical for the successful CD to admicelle ME of analytes. The SEFs obtained were comparable to previously developed off-line microextraction techniques; however, our in-line approach is much faster. Off-line microextraction takes about 20–40 min, while CD to admicelle ME takes only 8.5 min or ~ 30 min from preconditioning to analysis (see Table 1). Finally, the potential of CD to admicelle ME for use in real samples was demonstrated in the determination of anionic analytes in artificial urine.

## Supplementary Information

Below is the link to the electronic supplementary material.

Supplementary file1 (DOCX 723 KB)
