# Supplementary material for: In-line sample concentration in capillary electrophoresis by cyclodextrin to admicelle microextraction
Source: Anal Bioanal Chem. 2022 Aug 18;414(22):6671–80. doi: 10.1007/s00216-022-04230-0 (PMC9411250; doi:10.1007/s00216-022-04230-0)
Supplement: Supplementary file 1 — Supplementary file1 (DOCX 723 KB) [file 216_2022_4230_MOESM1_ESM.docx]

***Analytical and Bioanalytical Chemistry***

**In-line sample concentration in capillary electrophoresis by cyclodextrin to admicelle microextraction**

Andaravaas Patabadige Jude Prasanna Vaas, Raymond B. Yu and Joselito P. Quirino*

Australian Centre for Research on Separation Science (ACROSS), School of Natural Sciences-Chemistry,

University of Tasmania, Australia 7001

*correspondence to: [jquirino@](mailto:jquirino@)utas.edu.au

**This Supporting Information (SI) file includes:**

SI Fig. S1. Sample concentration of model anionic analytes by P^2^ME (cyclodextrin to admicelles ME) using various anionic CDs

……………….. (page 2)

SI Fig. S2. Effect of CD concentration on stacking

……………….. (page 3)

SI Fig. S3. Initial studies on the effect of CD plug length on stacking

……………….. (page 4)

SI Fig, S4. Sample injection time versus sensitivity enhancement factor (SEF) using sample:CD plug of 1:4

……………….. (page 5)

SI Fig. S5. Sample injection time versus sensitivity enhancement factor (SEF) using sample:CD plug of 1:2

……………….. (page 6)


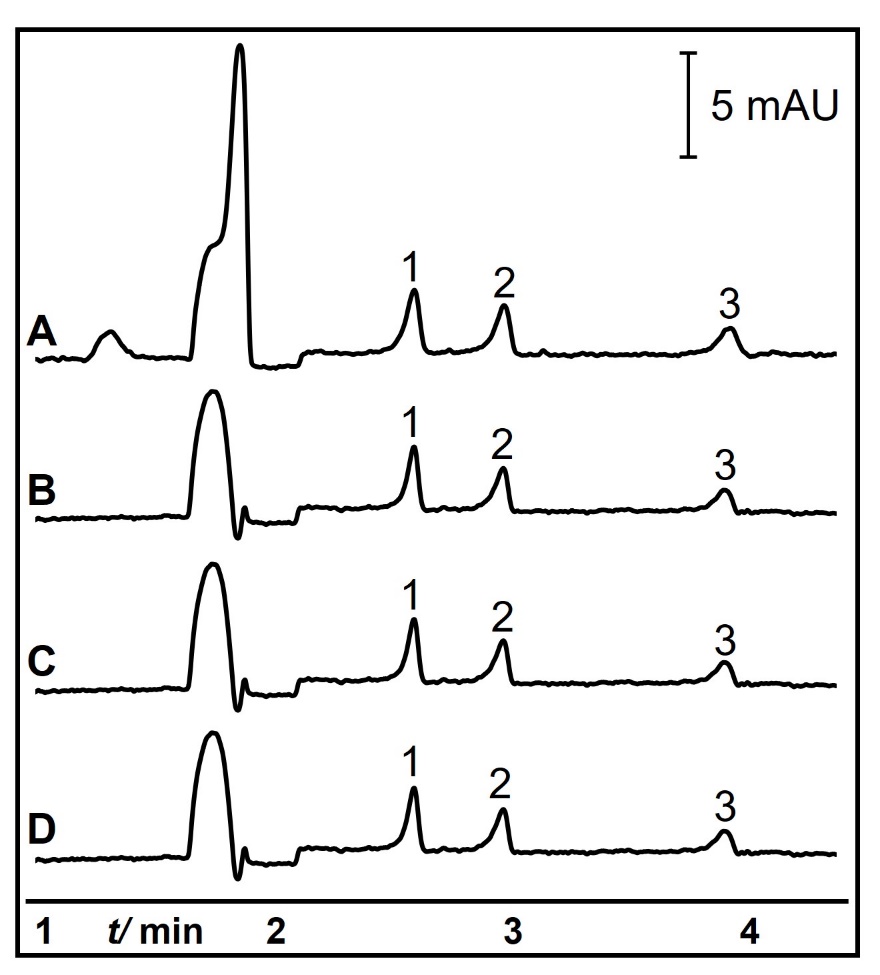


SI Fig. S1. Sample concentration of model anionic analytes by P^2^ME (cyclodextrin to admicelles ME) using various anionic CDs. The CDs used were sulphated α-CD (a), sulphated γ-CD (b), γ-CD phosphate sodium salt (c) and carboxymethyl-β-CD (d). The CD plug was 2 mM CD in 20 mM sodium tetraborate (pH 9.2). Analyte concentration, sample diluent and identification, P^2^ME injection program and capillary dimension were as in Fig. 3. Other conditions are mentioned in Materials and methods.


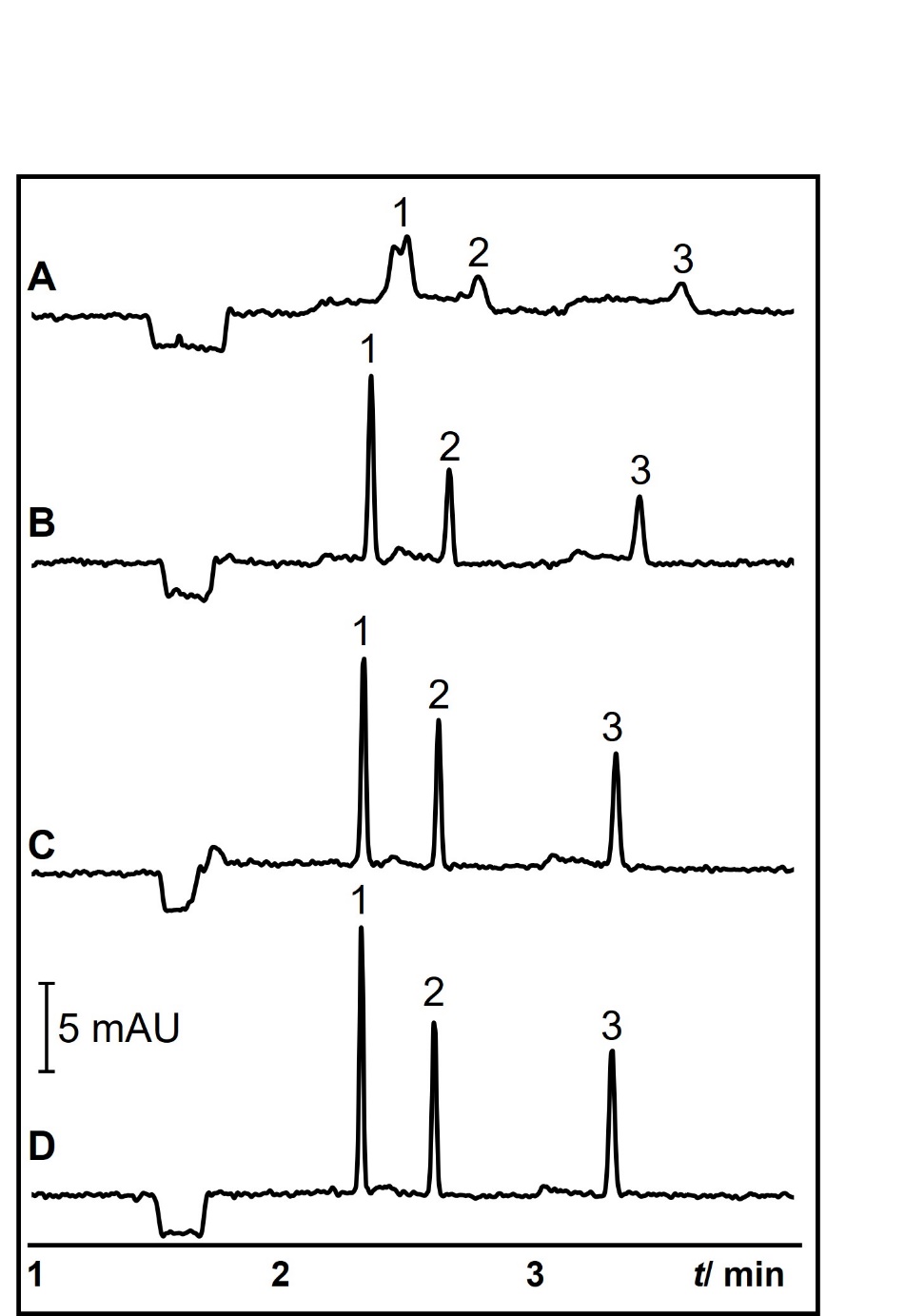


SI Fig. S2. Effect of CD concentration on sample concentration. The CD plug was α-CD in 20 mM sodium tetraborate (pH 9.2). α-CD concentrations used were 0.05 mM (a), 0.1 mM (b), 2 mM (c) and 20 mM (d). Analyte concentration, sample diluent and identification, P^2^ME (CD to admicelles ME) injection program and capillary dimension were as Fig. 3. Capillary dimensions were 37.5 cm (29 cm from inlet to UV detector) x 50 µm i.d. Other conditions are mentioned in Materials and methods.


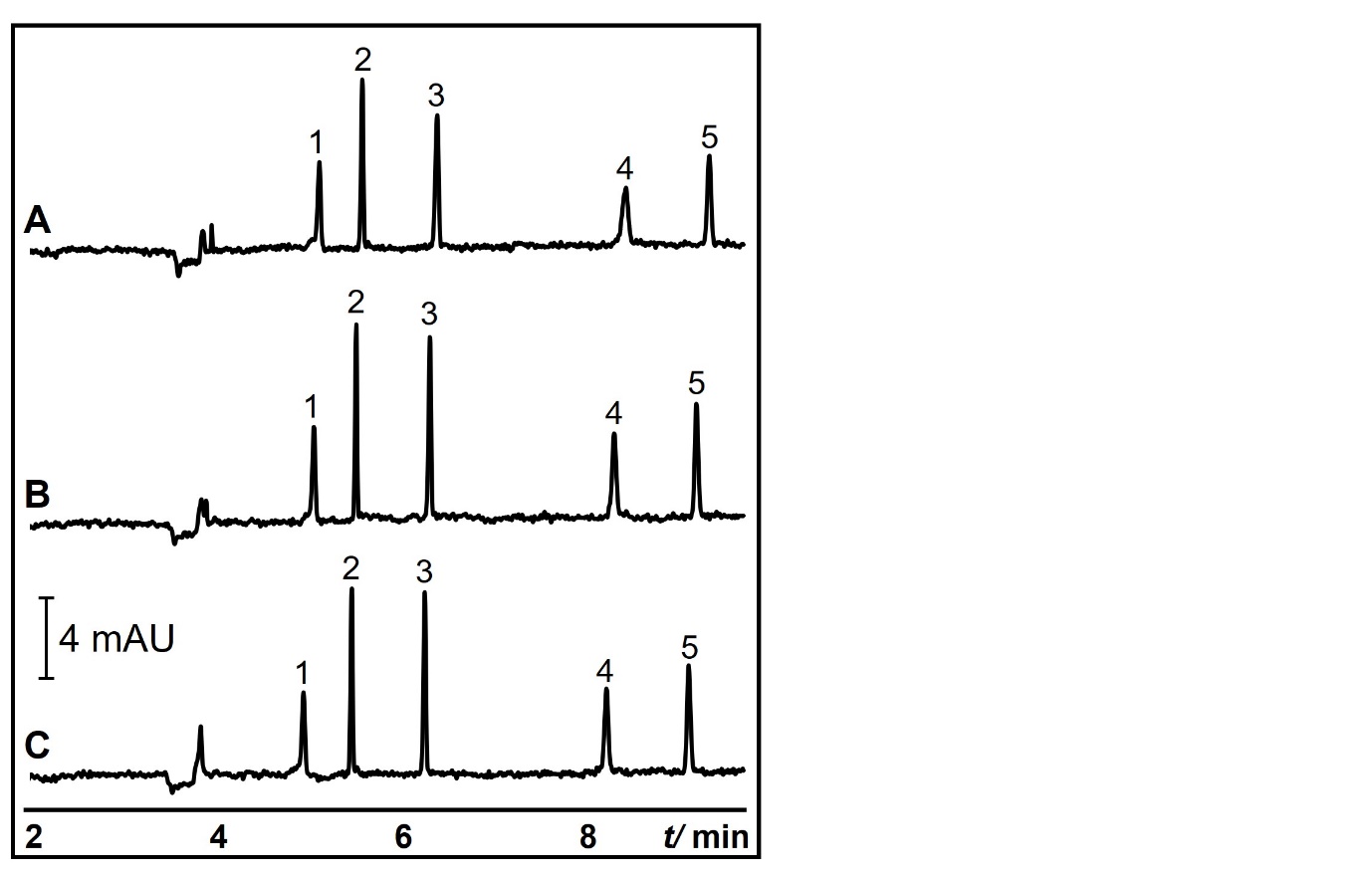


SI Fig. S3. Initial studies on the effect of CD plug length on sample concentration. The CD plug was 20 mM α-CD in 20 mM sodium tetraborate (pH 9.2). Model anionic analytes were 4 µg/mL each of 4-bromophenol (1), sulindac (2), sulfamethizole (3), 4-vinylbenzoic acid (4) and succinylsulfathiazole (5) in 0.2 mM CTAB in 20 mM sodium tetraborate (pH 9.2). P^2^ME (cyclodextrin to admicelles ME) injection program was as follows: injection of 0.2 mM CTAB in 20 mM sodium tetraborate (pH 9.2) at 50 mbar for 15 sec, sample injection at 50 mbar for 20 sec, and injection of CD plug at 50 mbar for 1 sec (a), 5 sec (b) or 10 sec (c). Capillary dimensions were 50 cm (41.5 cm from inlet to UV detector) x 50 µm i.d. Other conditions are mentioned in Materials and methods.


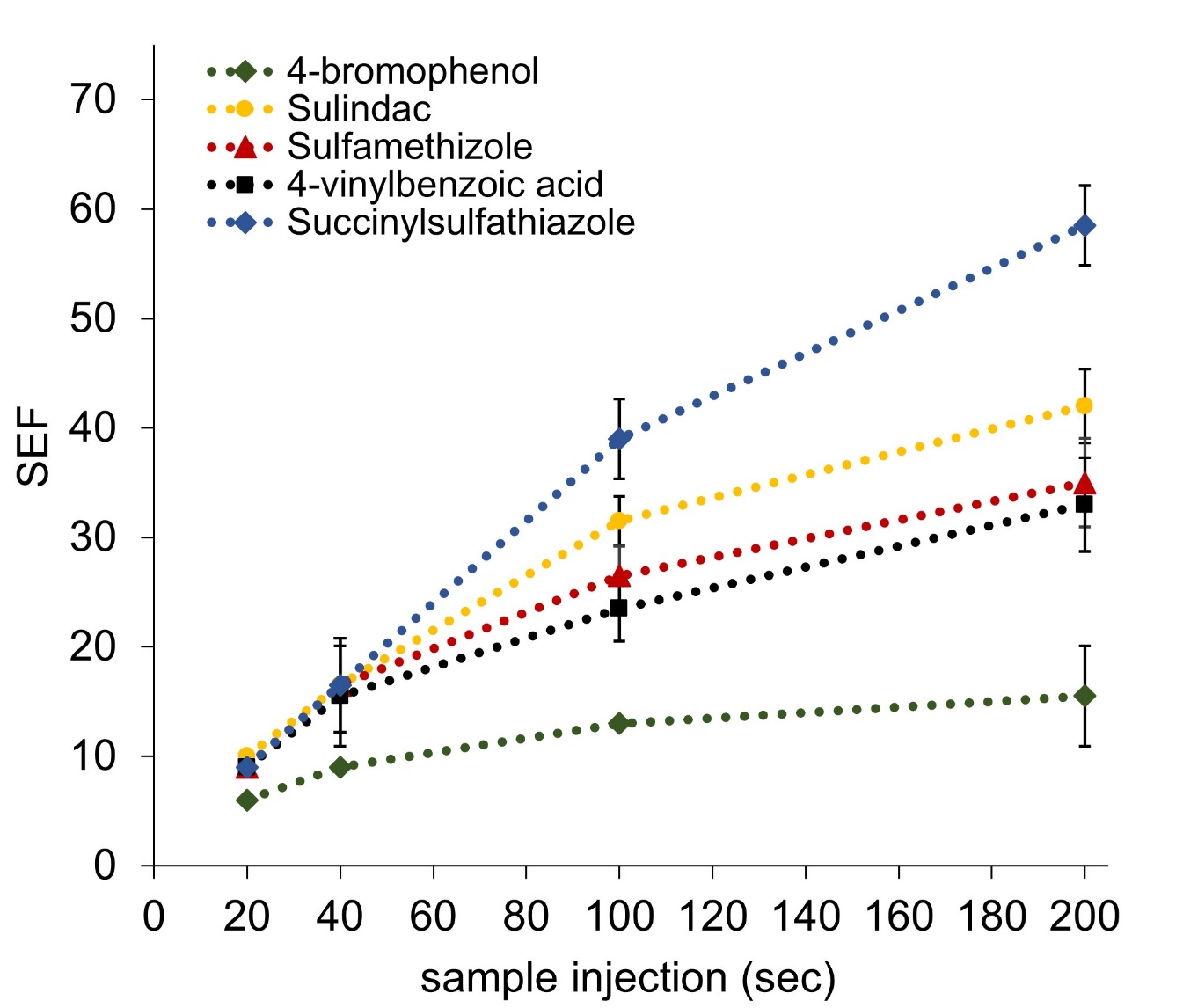


SI Fig. S4. Sample injection time versus sensitivity enhancement factor (SEF) using CD:sample of 1:4. CD plug, analyte concentration and diluent, and capillary dimensions are as in SI Fig. S3. Prior to sample injection, a solution of 0.2 mM CTAB in 20 mM sodium tetraborate (pH 9.2) was injected into the capillary at 50 mbar for 15 sec. Sample injection and injection of CD plug were performed at 50 mbar. The time of the CD plug injection was one-half of sample injection time. Other conditions are mentioned in Materials and methods.


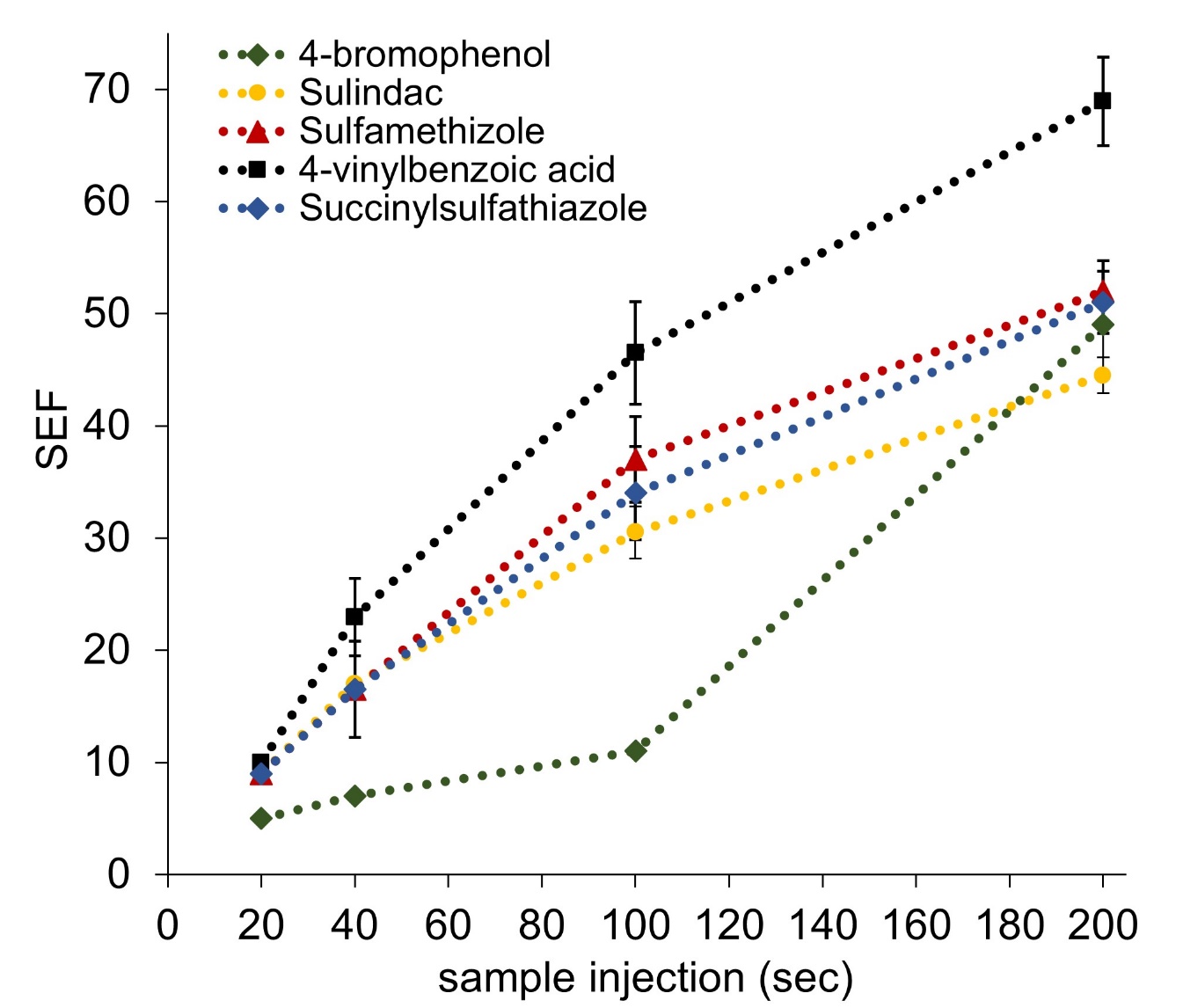


SI Fig. S5. Sample injection time versus SEF using CD:sample of 1:2. CD plug, analyte concentration and diluent, and capillary dimensions are as in SI Fig. S3. Prior to sample injection, a solution of 0.2 mM CTAB in 20 mM sodium tetraborate (pH 9.2) was injected into the capillary at 50 mbar for 15 sec. Sample injection and injection of CD plug were performed at 50 mbar. The time of the CD plug injection was one-half of sample injection time. Other conditions are mentioned in Materials and methods.
